# Supplementary material for: Anticipatory postural control emerges from a predictive and optimized strategy for movement preparation
Source: Commun Biol. 2026 Apr 6;9:629. doi: 10.1038/s42003-026-10016-2 (PMC13161268; doi:10.1038/s42003-026-10016-2)
Supplement: Supplementary file 2 — Supplementary Information [file 42003_2026_10016_MOESM2_ESM.pdf]

Supplementary Information for

Anticipatory Postural Control Emerges from a Predictive and Optimized Strategy for Movement Preparation

Tetsuro Funato, Miho Ogawa, Akira Konosu, Dai Yanagihara

Tetsuro Funato
Email: funato@uec.ac.jp

This PDF file includes:

Supplementary Methods
Figures S1 to S6
Tables S1 to S7
Supplementary References

Supplementary Methods

Body Model. The body model consists of two links with two joints at the ankle and hip and is modeled so that changes in the floor angle act as external forces. The equations of motion are as follows.

$$\boldsymbol{M}\ddot{\boldsymbol{\theta}} + \boldsymbol{G}g + \boldsymbol{C} = \boldsymbol{\tau} + \boldsymbol{\tau_{noise}} \tag{1}$$

Inertia term (mass term)

$$\boldsymbol{M} = \begin{bmatrix} M_{11} & M_{12} \\ M_{12} & M_{22} \end{bmatrix} \tag{2}$$

$$M_{11} = J_1 + J_2 + h_1^2 m_1 + h_2^2 m_2 + 2\cos\theta_2\, h_2 l_1 m_2 + l_1^2 m_2$$
$$M_{12} = J_2 + h_2^2 m_2 + \cos\theta_2\, h_2 l_1 m_2$$
$$M_{22} = J_2 + h_2^2 m_2$$

Nonlinear terms (centrifugal and Coriolis terms)

$$\boldsymbol{C} = \begin{bmatrix} C_1 \\ C_2 \end{bmatrix} \tag{3}$$

$$\begin{aligned} C_1 = &-2m_2\, l_1\, h_2\, \dot{\theta}_1\, \dot{\theta}_2\, \sin\theta_2 - m_2\, l_1\, h_2\, \dot{\theta}_2^2\, \sin\theta_2 \\ &-2m_2\, l_1\, h_2\, \dot{\theta}_2\, \sin\theta_2\, \dot{\theta}_f \\ &+\ddot{\theta}_f (J_1 + J_2 + h_1^2\, m_1 + h_2^2\, m_2 + 2\cos\theta_2\, h_2 l_1 m_2 + l_1^2\, m_2) \end{aligned}$$
$$\begin{aligned} C_2 = &\, m_2 l_1 h_2\, \dot{\theta}_1^2\, \sin\theta_2 + 2m_2 l_1 h_2\, \dot{\theta}_1\, \sin\theta_2\, \dot{\theta}_f \\ &+m_2 l_1 h_2\, \sin\theta_2\, \dot{\theta}_f^2 + \ddot{\theta}_f\, (J_2 + h_2^2 m_2 + \cos\theta_2\, h_2 l_1 m_2) \end{aligned}$$

Gravitational term

$$\boldsymbol{G} = \begin{bmatrix} G_1 \\ G_2 \end{bmatrix} \tag{4}$$

$$G_1 = -m_1 h_1 \sin(\theta_1 + \theta_f) - m_2 h_2 \sin(\theta_1 + \theta_2 + \theta_f) - m_2 l_1 \sin(\theta_1 + \theta_f)$$
$$G_2 = -m_2 h_2 \sin(\theta_1 + \theta_2 + \theta_f)$$

Here, $\boldsymbol{\theta} = [\theta_1, \theta_2]^{\mathrm{T}}$. The ankle joint angle θ_1 is the angle relative to the perpendicular to the floor, and the hip joint angle θ_2 is the angle relative to the lower body (Supplementary Figure S5). m_1 and m_2 are the masses of the lower and upper body, respectively, and l_1 and l_2 are length of the lower and upper body, h_1 and h_2 are the distance from the joint to the COM of the lower and upper body. J_1 and J_2 are the moments of inertia

around the COM of the lower and upper body, g is acceleration due to gravity. $\boldsymbol{\tau} = [\tau_1, \tau_2]^{\mathrm{T}}$, where τ_1 and τ_2 are joint torques. The noise term $\boldsymbol{\tau}_{\mathbf{noise}}$ is the torque due to biological noise and is modeled as Gaussian white noise with a magnitude of 0.33 Nm[1], generated as separate series for the hip and ankle, respectively. The values of each body parameter were taken from a quiet standing study using a two-link body model[2] (Supplementary Table S7A).

Muscle Model. Four muscles are arranged in the musculoskeletal model: the tibialis anterior (TA) and gastrocnemius (GC) muscles around the ankle, and the iliopsoas (IL) and gluteus maximus (GM) muscles around the hip. Control inputs are generated as muscle activity levels and converted into torque through the dynamic properties of the muscles, resulting in physical effects on the body. The dynamic muscle model consists of contraction elements (CE) that contract in response to control inputs and passive elastic elements (PE), and generates muscle force according to the force-length and force-velocity relationships in these elements, using the following mathematical model[3, 4].

$$F_m = \overline{F}_m^{CE} \cdot k\left(\frac{L_m}{\overline{L}_m}\right) \cdot h\left(\frac{\dot{L}_m}{\bar{\dot{L}}_m}\right) y_m + F_m^{PE} \tag{5}$$

$$F_m^{PE} = 0.0159\overline{F}_m^{CE} \exp\left(5.9\overline{L}_m\left(\frac{L_m}{\overline{L}_m} - 1\right) - 1\right)$$

where F_m is the muscle force, $\overline{F}_m^{CE}$ is the isometric maximum contractile force, $\overline{L}_m$ is the natural length. $\bar{\dot{L}}_m$ is the maximum contraction velocity, which was set to 3.0 m/s for all muscles. y_m is muscle activity level and is generated by model predictive control. The force-length relationship $k(L_m/\overline{L}_m)$ and force-velocity relationship $h(\dot{L}_m/\bar{\dot{L}}_m)$ are expressed by the following equations using normalized muscle length $\xi_m = L_m/\overline{L}_m$ and normalized muscle contraction velocity. $\eta_m = \dot{L}_m/\bar{\dot{L}}_m$.

$$k(\xi_m) = 0.32 + 0.71\exp\{-1.112(\xi_m - 1)\} \cdot \sin\{3.722(\xi_m - 0.656)\}$$
$$h(\eta_m) = \{1 + \tanh(3.0\eta_m)\}$$

Joint torque is calculated by multiplying the muscle force F_m with the moment arm. The values of each muscle parameter are shown in Supplementary Table S7B.

Effect of Floor Tilt. The effect of the change in the floor tilt angles θ_f, the body appears as a function of the floor angular velocity $\dot{\theta}_f$ and the floor angular acceleration $\ddot{\theta}_f$ in the

centrifugal and Coriolis terms (equation (3)). Here, in eq. (3) the coefficients of $\ddot{\theta}_f$ in C_1 and C_2 are equal to M_{11} and M_{12}, respectively. Then, if we divide $\boldsymbol{C}$ into the $\ddot{\theta}_f$ term $\boldsymbol{C_f}\ddot{\theta}_f = [M_{11} \quad M_{12}]^{\mathrm{T}}\ddot{\theta}_f$ and the other terms $\widetilde{\boldsymbol{C}}(\theta_2, \dot{\theta}_1, \dot{\theta}_2, \dot{\theta}_f)$, the equation becomes as follows.

$$\boldsymbol{M}\ddot{\boldsymbol{\theta}} + \boldsymbol{G}g + \widetilde{\boldsymbol{C}}(\theta_2, \dot{\theta}_1, \dot{\theta}_2, \dot{\theta}_f) + \boldsymbol{C_f}\ddot{\theta}_f = \boldsymbol{\tau}$$
$$\ddot{\boldsymbol{\theta}} = \boldsymbol{M^{-1}}(-\boldsymbol{G}g - \widetilde{\boldsymbol{C}} + \boldsymbol{\tau}) - \boldsymbol{M^{-1}C_f}\ddot{\theta}_f$$

Furthermore, since $\boldsymbol{M^{-1}C_f} = \boldsymbol{M^{-1}}[M_{11} \quad M_{12}]^{\mathrm{T}} = [1\ 0]^{\mathrm{T}}$,

$$\ddot{\boldsymbol{\theta}} = \boldsymbol{M^{-1}}(-\boldsymbol{G}g - \widetilde{\boldsymbol{C}} + \boldsymbol{\tau}) - \begin{bmatrix}1\\0\end{bmatrix}\ddot{\theta}_f \quad (6)$$

This means that the floor acceleration $\ddot{\theta}_f$ acts directly only on the ankle acceleration $\ddot{\theta}_1$, i.e., the $\ddot{\theta}_f$ term acts as if the floor inclination changes the angle of the ankle as it does (note that the floor velocity term $\dot{\theta}_f$ also acts as the $\widetilde{\boldsymbol{C}}(\theta_2, \dot{\theta}_1, \dot{\theta}_2, \dot{\theta}_f)$ term).

Stiffness Control. A control element contributing to stability during standing includes the passive effect of body stiffness, which plays a stabilizing role in cooperation with the stretch reflex[5]. This mechanism generates the following torques at the ankle and hip, respectively:

$$\tau_{\mathrm{stiff}} = -K_{a,h}(\theta - \hat{\theta}) - B_{a,h}\,\dot{\theta}$$

The values of K_a, K_h, B_a, and B_h, which correspond to ankle and hip stiffness and viscosity, were set based on previous literature[2]. Note that K_a values of 0.7 to $0.8 \times mgh$ were used in past standing model studies, while measurements of the muscles around the ankle using an ergometer[6] showed lower values. Therefore, in this study, we examined the simulation behavior in the range of 0.1 to $0.8 \times mgh$ (Supplementary Figure S6). The results showed that control by GC activity, as observed in human experiments, was observed when the K_a value was $0.3 \times mgh$ or less, and control by TA activity was observed when the value was $0.4 \times mgh$ or higher. Therefore, we decided to proceed with the analysis using $K_a = 0.3 \times mgh$. The values of these control parameters are summarized in Supplementary Table S7C.

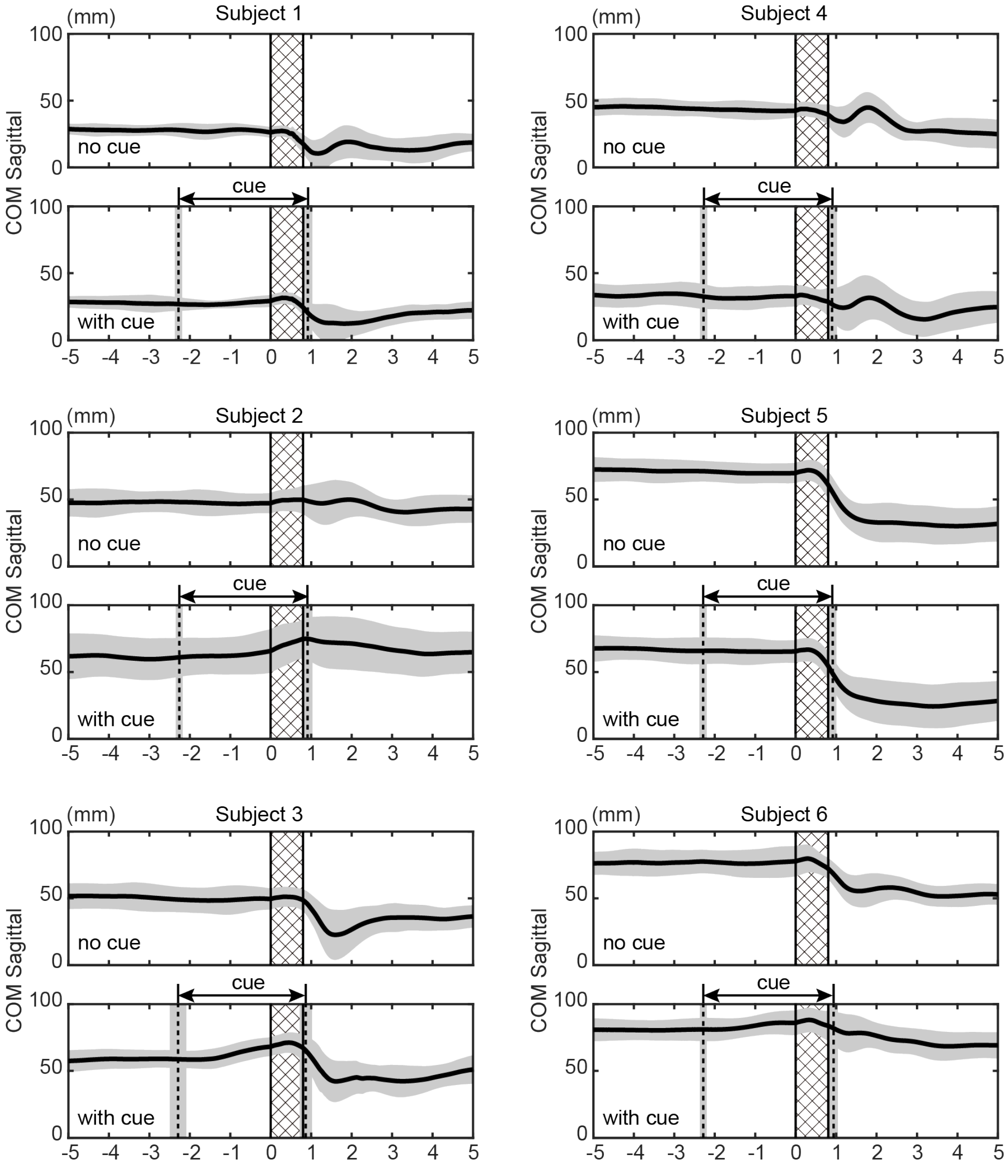

Subject 1
Subject 2
Subject 3
Subject 4
Subject 5
Subject 6
(mm)
COM Sagittal
no cue
with cue
cue

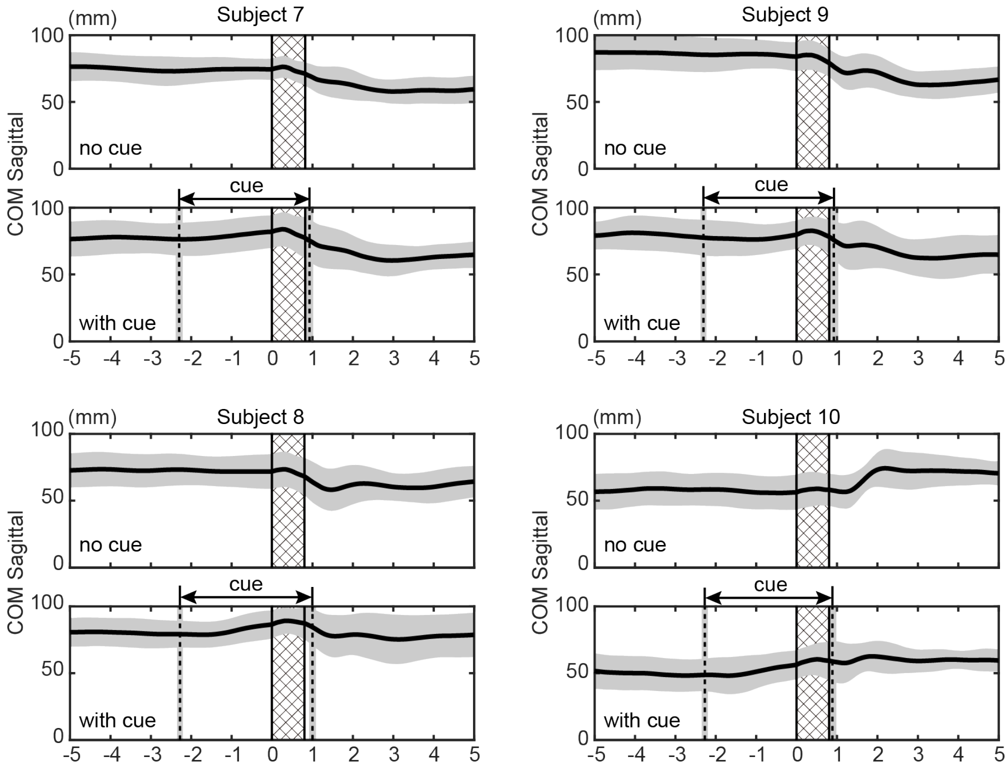

Fig. S1. Results of COM time series for all subjects obtained in the experiment. The anteroposterior position of the COM is shown with the ankle position as 0 mm. The results of 20 trials for each subject (excluding the first 10 trials in each condition) are shown as the mean (solid line) and standard deviation (SD; gray area) in each condition (n = 20 trials per condition). The slanted area represents the floor tilt period, and the dotted line (area behind the dotted line) in the trials with a cue indicates the mean (SD) of the start and stop times of the cue sound.

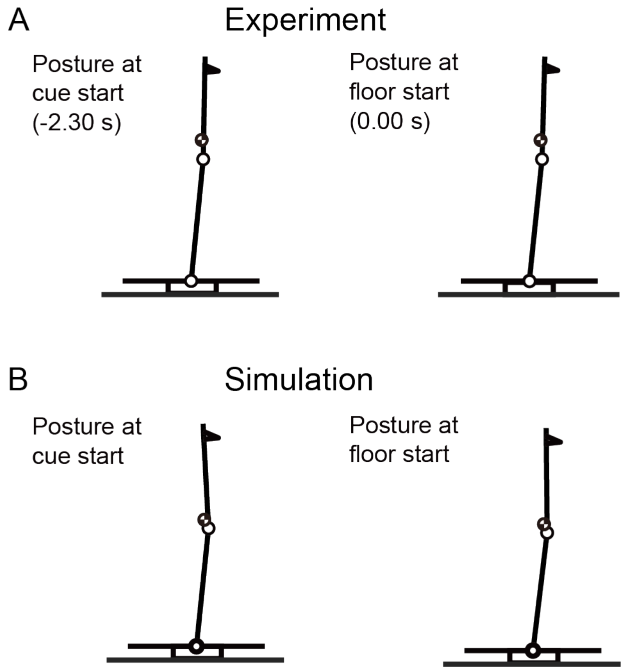

Fig. S2. Average posture at cue start (CS) and floor start (FS). (A) result of the experiment for the same subject as Fig. 2ACE. **(B)** result of the simulation.

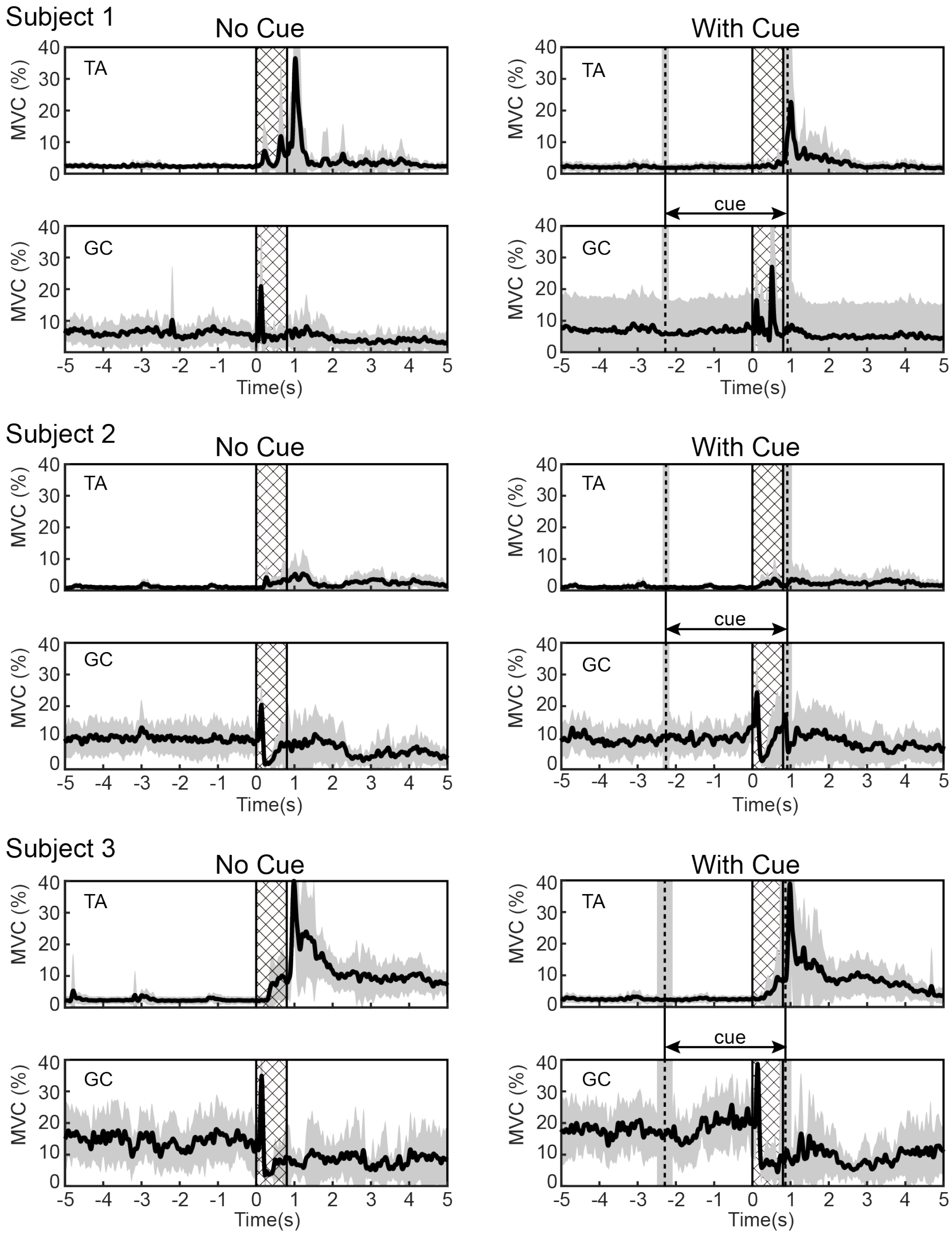

Subject 1
No Cue
With Cue
TA
GC
cue
MVC (%)
Time(s)
Subject 2
No Cue
With Cue
TA
GC
cue
MVC (%)
Time(s)
Subject 3
No Cue
With Cue
TA
GC
cue
MVC (%)
Time(s)

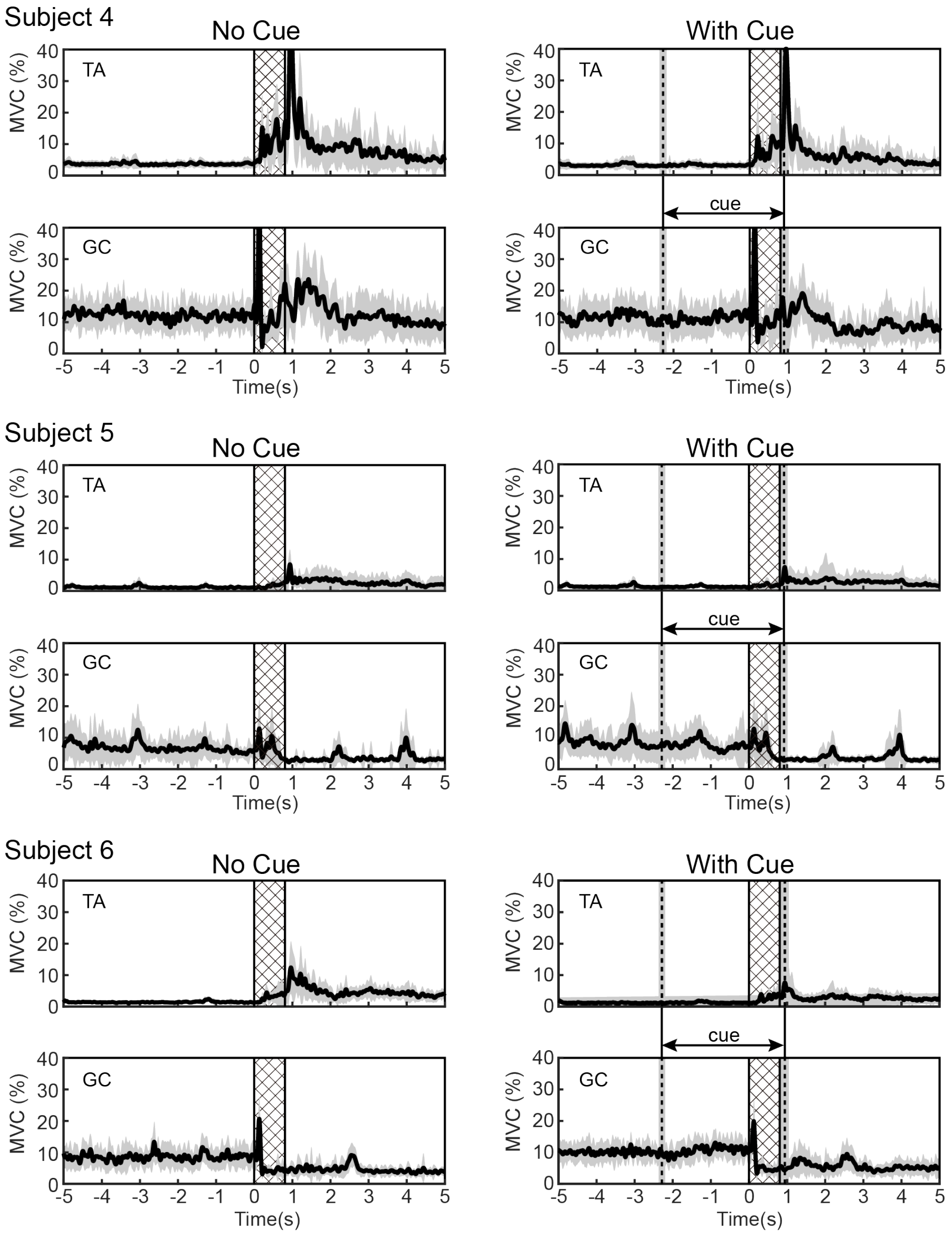
Subject 4
No Cue
With Cue
TA
GC
MVC (%)
cue
Time(s)
Subject 5
No Cue
With Cue
TA
GC
MVC (%)
cue
Time(s)
Subject 6
No Cue
With Cue
TA
GC
MVC (%)
cue
Time(s)

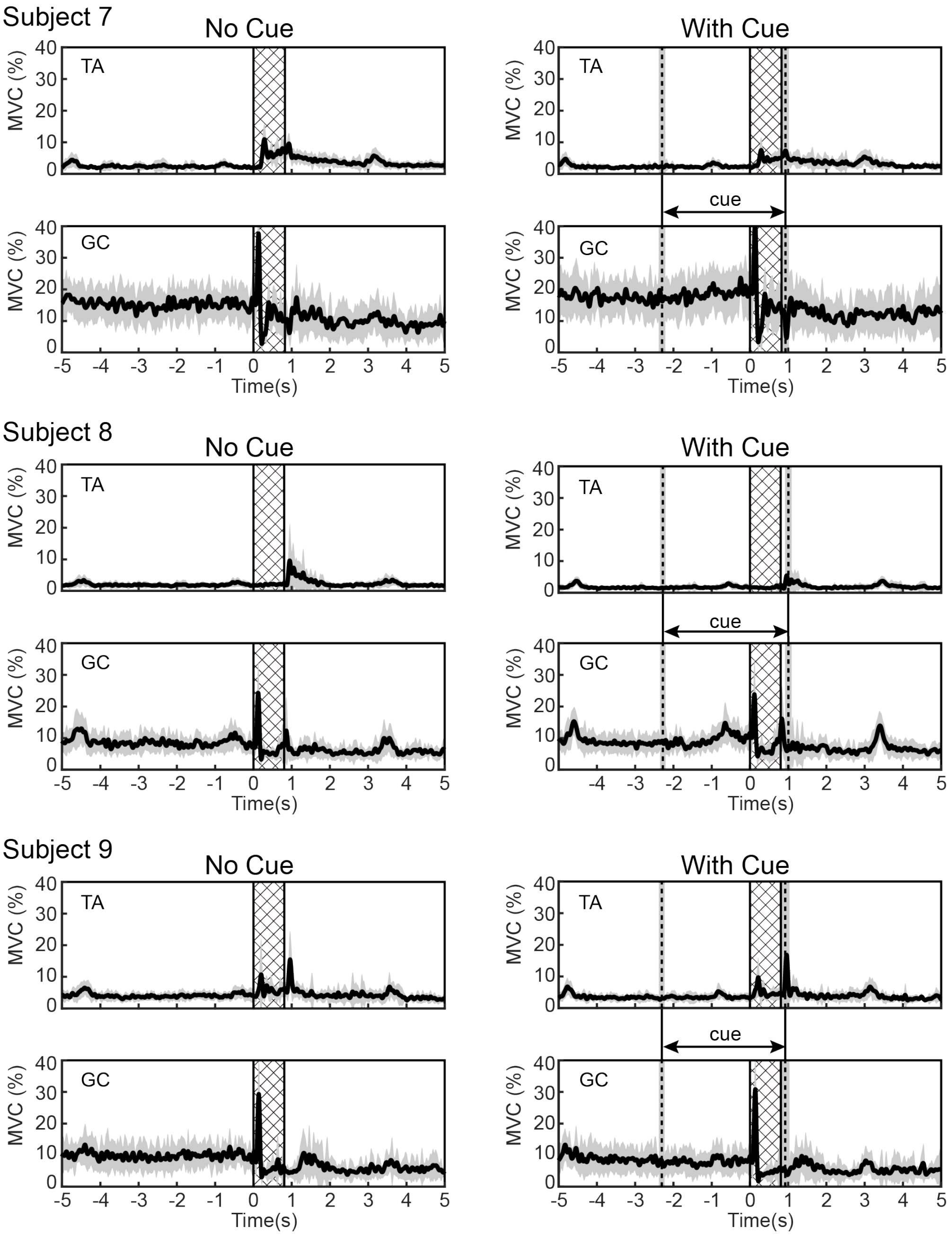
Subject 7
No Cue
With Cue
TA
GC
MVC (%)
cue
Time(s)
Subject 8
No Cue
With Cue
TA
GC
MVC (%)
cue
Time(s)
Subject 9
No Cue
With Cue
TA
GC
MVC (%)
cue
Time(s)

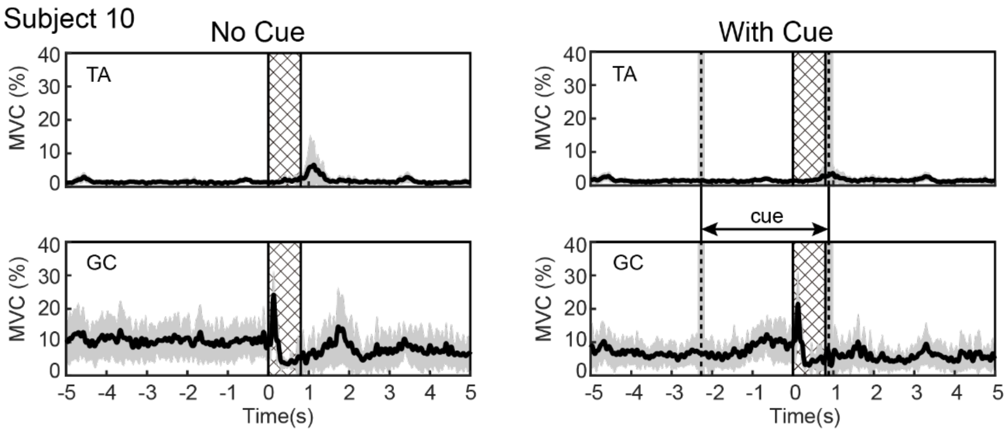

Fig. S3. Results of EMG time series for all subjects obtained in the experiment. TA: tibialis anterior; GC: gastrocnemius. The results of 20 trials for each subject (excluding the first 10 trials in each condition) are shown as mean (solid line) and standard deviation (SD; gray area) in each condition (n = 20 trials per condition). The slanted area represents the floor tilt period, and the dotted line (area behind the dotted line) in the trials with a cue indicates the mean (SD) of the start and stop times of the cue sound.

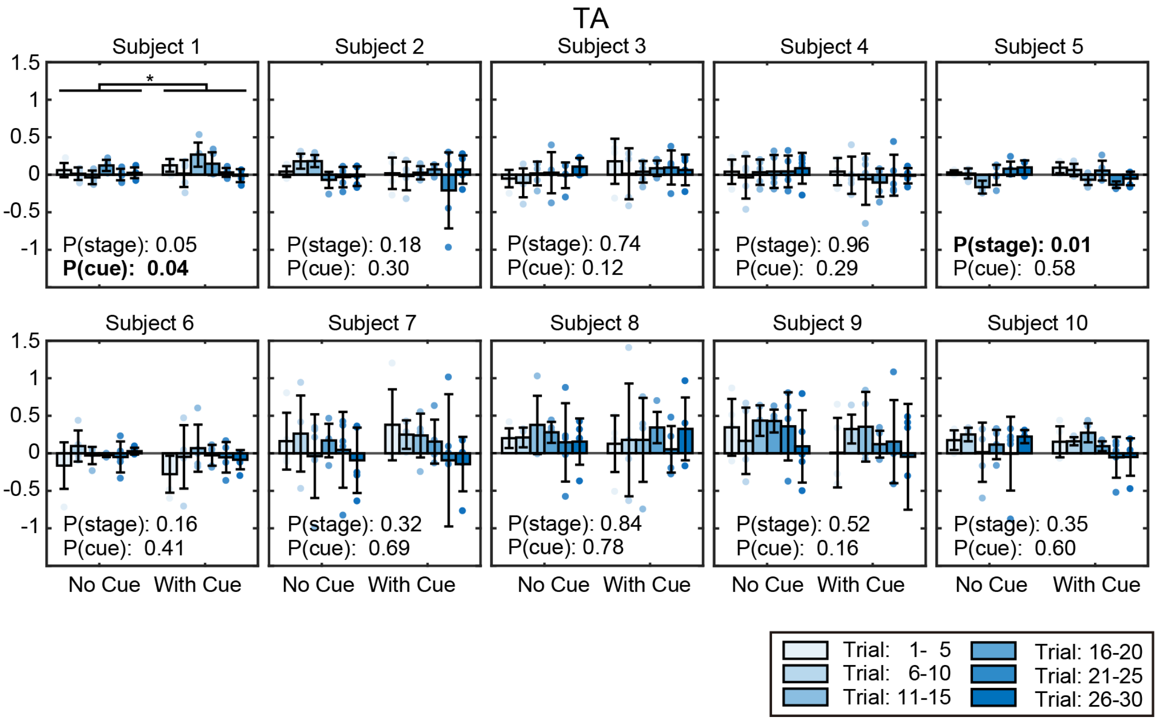

Fig. S4. Effect of trial stage on tibialis anterior (TA) activity changes during the cue start (CS)-floor start (FS) period. Each condition (no cue and cue) consists of 30 trials divided into 6 stages of 5 trials each. For each subject, values in each stage are shown as the mean and SD across the trials within that stage (n=5 trials per stage). The *p* values in the figure represent the results of a two-way ANOVA for trial stage and cue presence. Significant differences due to cue are indicated by * in the figure. *: $p < 0.05$.

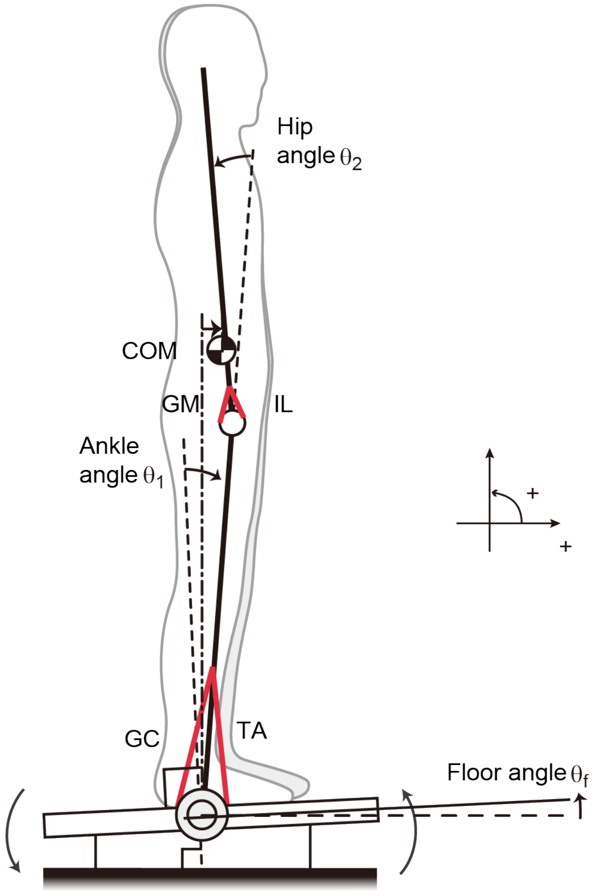

Fig. S5. Definition of the body model for simulation. The body model consists of two links with two joints at the ankle and hip, and changes in the floor angle act as external forces. Four muscles are included in the muscle model: the tibialis anterior (TA) and gastrocnemius (GC) muscles around the ankle, and the iliopsoas (IL) and gluteus maximus (GM) muscles around the hip. The floor tilt angle, ankle angle, and hip angle are defined as the relative angles from the horizontal plane, the angle perpendicular to the floor, and the angle from the lower body, respectively. COM is defined as the anteroposterior displacement, with the ankle position as 0.

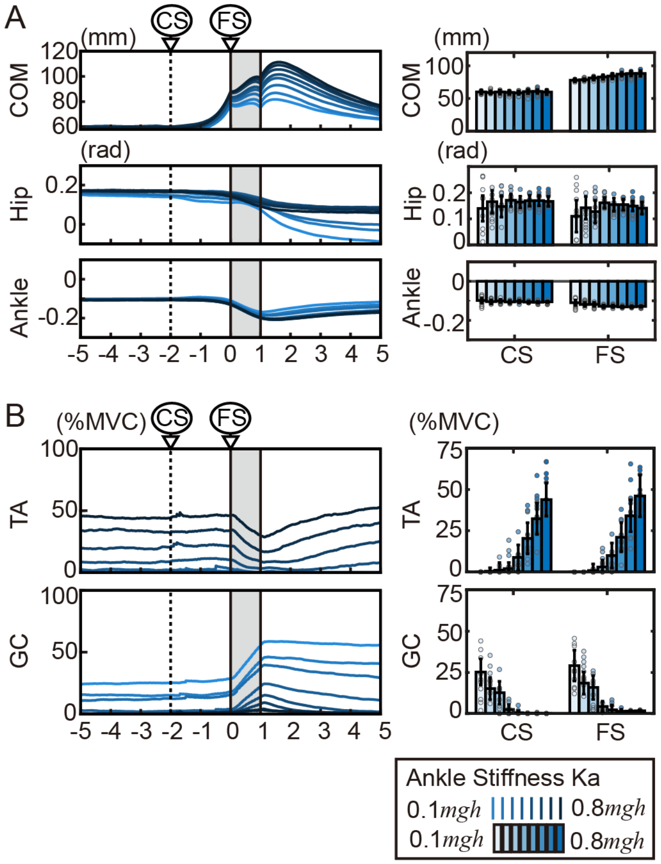

Fig. S6. Effect of the ankle stiffness *Ka* on predictive behavior in the simulation. The results of simulations in which the ankle stiffness *Ka* was changed at equal intervals from 0.1*mgh* to 0.8*mgh* are shown. Each result shows the average of 20 trials performed with different random noise. **(A)** Results for COM, hip joint, and ankle joint. The time at which the tilt became predictable (CS) and the start time of floor tilting (FS). **(B)** Results for muscle activity. TA: tibialis anterior; GC: gastrocnemius. All values are presented as the mean and SD (n = 20 independent simulation trials).

Table S1. Statistical values for the significance of changes in movement and muscle activity during the cue period. Results for *p*-values, *t*-values, and degrees of freedom (df) for **(A)** movement (COM, hip, ankle) and **(B)** muscle activity (TA: tibialis anterior; GC: gastrocnemius). Bold *p*-values indicate significance at $p < 0.05$.

A

COM			Hip			Ankle		
p-value	*t*-value	df	*p*-value	*t*-value	df	*p*-value	*t*-value	df
**0.020**	-2.43	38	**0.020**	-2.42	38	**0.003**	3.16	38
**0.009**	-2.74	38	0.396	-0.86	38	**0.011**	2.66	38
**0.002**	-3.41	37	**0.050**	2.03	37	**0.003**	3.16	37
0.389	-0.87	38	0.357	0.93	38	0.614	0.51	38
0.587	-0.55	38	0.397	-0.86	38	0.389	0.87	38
**0.002**	-3.35	36	0.505	0.67	36	**0.001**	3.70	36
**0.049**	-2.03	38	0.203	1.30	38	**0.031**	2.24	38
**0.000**	-6.32	38	0.128	1.55	38	**0.000**	6.51	38
0.207	-1.28	37	0.090	1.74	37	0.158	1.44	37
**0.000**	-4.08	38	0.257	-1.15	38	**0.000**	5.66	38

B

TA			GC		
p-value	*t*-value	df	*p*-value	*t*-value	df
0.066	-1.89	38	0.059	-1.94	38
0.720	0.36	38	0.419	-0.82	38
0.672	-0.43	37	0.087	-1.76	37
0.138	1.52	38	0.354	-0.94	38
0.217	1.25	38	0.915	0.11	38
0.863	0.17	38	**0.046**	-2.06	38
0.990	0.01	38	**0.021**	-2.40	38
0.904	0.12	38	**0.003**	-3.18	38
0.154	1.45	37	0.547	-0.61	37
0.821	0.23	38	**0.005**	-2.95	38

Table S2. Results of ANOVA tests for COM displacement during the cue period. (A) Results of two-way ANOVA by trial stage and cue presence. **(B)** Results of one-way ANOVA testing significant differences by trial stage separately for No Cue and With Cue conditions. Bold text indicates results with $p < 0.05$.

A

Subject	1		2		3		4		5	
	stage	cue	stage	cue	stage	cue	stage	cue	stage	cue
p-value	0.58	**0.05**	0.50	**0.00**	0.52	**0.00**	0.17	0.34	**0.01**	0.13
F-value	0.77	4.09	0.89	14.20	0.85	18.70	1.62	0.93	3.36	2.35
df	5	1	5	1	5	1	5	1	5	1

Subject	6		7		8		9		10	
	stage	cue	stage	cue	stage	cue	stage	cue	stage	cue
p-value	0.44	**0.00**	0.05	**0.04**	0.16	**0.00**	0.18	0.16	**0.02**	**0.00**
F-value	0.97	16.70	2.39	4.67	1.65	56.70	1.58	2.02	2.89	40.50
df	5	1	5	1	5	1	5	1	5	1

B

No Cue

Subject	1	2	3	4	5	6	7	8	9	10
p-value	0.20	0.57	0.33	0.34	0.06	0.92	0.12	0.68	0.20	0.10
F-value	1.61	0.79	1.22	1.19	2.49	0.27	2.01	0.64	1.58	2.09
df	5	5	5	5	5	5	5	5	5	5

With Cue

Subject	1	2	3	4	5	6	7	8	9	10
p-value	0.40	0.14	0.72	0.29	**0.03**	0.24	0.20	0.14	0.45	**0.01**
F-value	1.07	1.83	0.58	1.31	3.15	1.47	1.59	1.88	0.99	4.20
df	5	5	5	5	5	5	5	5	5	5

Table S3. Results of ANOVA tests for gastrocnemius (GC) activity change during the cue period. (A) Results of two-way ANOVA by trial stage and cue presence. (**B)** Results of one-way ANOVA testing significant differences by trial stage separately for No Cue and With Cue conditions. (**C)** Results of linear regression analysis for changes in GC activity on stages under cue conditions. Bold text indicates results with $p < 0.05$.

A

Subject	1		2		3		4		5	
	stage	cue	stage	cue	stage	cue	stage	cue	stage	cue
p-value	0.18	0.16	**0.04**	0.06	0.77	**0.00**	0.81	0.14	0.09	0.46
F-value	1.59	2.07	2.55	3.56	0.51	9.81	0.45	2.29	2.04	0.54
df	5	1	5	1	5	1	5	1	5	1

Subject	6		7		8		9		10	
	stage	cue	stage	cue	stage	cue	stage	cue	stage	cue
p-value	0.13	**0.02**	0.39	0.15	0.14	**0.00**	0.36	0.70	**0.01**	**0.00**
F-value	1.82	6.16	1.07	2.17	1.76	22.80	1.13	0.16	3.56	24.20
df	5	1	5	1	5	1	5	1	5	1

B

No Cue

Subject	1	2	3	4	5	6	7	8	9	10
p-value	0.30	0.44	0.64	0.97	0.82	0.46	0.17	0.16	0.35	0.66
F-value	1.29	1.00	0.69	0.18	0.44	0.97	1.70	1.74	1.19	0.66
df	5	5	5	5	5	5	5	5	5	5

With Cue

Subject	1	2	3	4	5	6	7	8	9	10
p-value	**0.01**	**0.02**	0.64	0.56	**0.03**	0.15	0.22	0.36	0.91	**0.01**
F-value	3.67	3.25	0.69	0.81	2.90	1.80	1.54	1.15	0.30	3.67
df	5	5	5	5	5	5	5	5	5	5

C

Subject	1	2	3	4	5	6	7	8	9	10
Coeff	-0.23	-0.67	-0.78	-0.30	-0.97	0.00	0.22	0.08	0.05	-0.24
p-value	0.19	**0.04**	0.17	0.27	**0.02**	0.99	0.30	0.63	0.77	0.52

Table S4. Results of ANOVA tests for tibialis anterior (TA) activity change during the cue period. Results of a two-way ANOVA with trial stage and cue presence as factors are shown.

Subject	1		2		3		4		5	
	stage	cue	stage	cue	stage	cue	stage	cue	stage	cue
p-value	0.05	**0.04**	0.18	0.30	0.74	0.12	0.96	0.29	**0.01**	0.58
F-value	2.38	4.32	1.60	1.11	0.54	2.50	0.20	1.16	3.77	0.32
df	5	1	5	1	5	1	5	1	5	1

Subject	6		7		8		9		10	
	stage	cue	stage	cue	stage	cue	stage	cue	stage	cue
p-value	0.16	0.41	0.32	0.69	0.84	0.78	0.52	0.16	0.35	0.60
F-value	1.68	0.68	1.21	0.16	0.41	0.08	0.85	2.06	1.15	0.27
df	5	1	5	1	5	1	5	1	5	1

Table S5. Time from tilt onset to peak gastrocnemius (GC) activity. Values (ms) represent the mean (SD) across 20 trials for each subject in the No Cue and With Cue conditions.

Subject	1	2	3	4	5
No Cue	94.7 (45.8)	101.2 (48.7)	73.2 (59.0)	66.2 (54.5)	72.7 (53.3)
With Cue	96.0 (36.9)	93.0 (44.7)	84.2 (59.7)	95.4 (55.4)	77.4 (51.7)

Subject	6	7	8	9	10
No Cue	78.0 (53.1)	97.4 (52.8)	92.5 (48.1)	84.4 (53.9)	69.5 (52.9)
With Cue	75.2 (50.1)	78.5 (53.8)	91.9 (44.5)	84.4 (59.3)	74.5 (51.4)

Table S6. Physical parameters of the subjects.

Subject number	Gender	Age	Body Length (m)	Body weight (kg)
1	female	31	1.56	69
2	female	25	1.58	62
3	female	51	1.73	60
4	female	49	1.56	51
5	female	45	1.52	44
6	male	46	1.64	66
7	male	48	1.72	74
8	male	29	1.69	60
9	male	57	1.70	75
10	male	34	1.76	65

Table S7. Parameters of the model for simulation. (A) Body parameters of the model. **(B)** Muscle parameters of the model. TA: tibialis anterior; GC: gastrocnemius; IP: iliopsoas; GM: gluteus maximus. Maximal isometric contraction force $\overline{\boldsymbol{F}}_{\boldsymbol{m}}^{\boldsymbol{CE}}$, Natural length $\overline{\boldsymbol{L}}_{\boldsymbol{m}}$, and moment arm (MA). **(C)** Control parameters of the model. $\boldsymbol{mgh}$ is the value determined by the mass $\boldsymbol{m}$, the acceleration of gravity $\boldsymbol{g}$, and the distance from the ankle to the COM $\boldsymbol{h}$.

A

Symbol	Description	Value
m_1	Mass of lower link (kg)	60*0.35
m_2	Mass of upper link (kg)	60*0.62
m	Mass of body (kg)	$m_1 + m_2$
l_1	Length of lower link (m)	1.70*0.51
l_2	Length of upper link (m)	1.70*0.45
h_1	Distance from distal end to lower link CoM (m)	1.70*0.255
h_2	Distance from distal end to upper link CoM (m)	1.70*0.225
h	Distance from ankle to COM (m)	$m_1 h_1 + m_2(l_1 + h_2)/m$
$\theta_1(0)$	Initial ankle angle (rad)	-0.11
$\theta_2(0)$	Initial hip angle (rad)	0.18

B

	$\overline{F}_m^{CE}$ (N)	$\overline{L}_m$ (N)	MA (m)
TA	1140	0.1	0.042
GC	1605	0.11	0.057
IP	1395	0.1	0.011
GM	1300	0.14	0.047

C

Symbol	Description	Value
	Control frequency for MPC (Hz)	100
N_P	Prediction Horizon (step)	30
N_M	Control Horizon (step)	2
w_u	Weight for control input	0.01
w_{Δ_u}	Weight for input change	1

Δ	Delay in sensory loop for MPC (ms)	150
K_a	Passive elastic coefficient at ankle	0.3× mgh
B_a	Passive viscosity coefficient at ankle (Nm s/rad)	4.0
K_h	Passive elastic coefficient at hip	0.4× mgh
B_h	Passive viscosity coefficient at hip (Nm s/rad)	10.0

Supplementary References

1. Funato, T., Aoi, S., Tomita, N. & Tsuchiya, K. Smooth enlargement of human standing sway by instability due to weak reaction floor and noise. *R Soc Open Sci.* **3**, 150570 (2016).
2. Suzuki, Y., Nomura, T., Casadio, M. & Morasso, P. Intermittent control with ankle, hip, and mixed strategies during quiet standing: a theoretical proposal based on a double inverted pendulum model. *J Theor Biol.* **310**, 55-79 (2012).
3. Ogihara, N. & Yamazaki, N. Estimation of human locomotory neural network from kinematic and kinetic data. *J Soc Biomechanisms.* **15**, 175-186 (2000).
4. Ogihara, N. & Yamazaki, N. Generation of human bipedal locomotion by a bio-mimetic neuro-musculo-skeletal model. *Biol Cybern.* **84**, 1-11 (2001).
5. Winter, D.A. *et al.* Stiffness control of balance in quiet standing. *J Neurophysiol.* **80**, 1211-1221 (1998).
6. Hof, A.L. In vivo measurement of the series elasticity release curve of human triceps surae muscle. *J Biomech.* **31**, 793-800 (1998).
